# Mercy Hospital

**Published:** 1870-07

**Authors:** 


### MERCY HOSPITAL.

THURSDAY, *June 10th, 1870.*

DROPSY.—Prof. Davis presented to the class an elderly man, robust, and in apparently good health. He complains of swelling in his face, some pain in his back and head, and of being easily tired during exertion. His legs are white, bloodless, hard and doughy, pitting readily upon pressure. The swelling commenced in the feet and ankles, about three months ago, and was gradually extending up the legs, while his strength was gradually declining. Position materially affects the swelling; the most dependent parts, whether face, back, hands, or legs, becoming œdematous. Hence, this is called general dropsy, to distinguish it from circumscribed, as when the fluid is contained in a cavity, or some locality exclusively.

If the fluid was contained in the cavity of the peritoneum, pericardium, pleura, ventricles of the brain, under the arachnoid, or in the air-cells of the lungs, or if it were confined to one limb, on account of some obstruction to the circulation, it would be local dropsy. When general dropsy affects the system, it must depend upon one of three causes, *viz.:* disease of the heart, kidney, or anæmia. This patient has had no malarial poison or hemorrhages, to affect and diminish the red corpuscles, and leave the blood in a watery condition. The history and appearance of the patient verify this conclusion. There is no history of trouble with the heart, neither does the stethoscope reveal any. An examination of the urine by the usual tests showed an abundance of albumen. The Prof. very carefully showed the tests to be used and the errors to be avoided. The facts here stated show that the dropsy, in this case, depends on disease of the kidneys; but they do not indicate clearly the nature of such disease. For the mere existence of albumen in the urine, even in large proportion, does not determine fully the nature of the morbid condition of the kidneys. It simply shows that something has altered the relation between the motion of the blood and the secreting cells of the kidney. Whether that alteration depends on temporary congestion, inflammatory irritation, or glandular degeneration must be determined by the history of the case, aided by microscopic examination of the urine. If the dropsical symptoms have come on rather suddenly, preceded by pain in the back, feverishness, and scanty urine, it may be regarded as almost certain, that the case is one of nephritic irritation or inflammation. Or, if the dropsy has come on more slowly, preceded and accompanied by some mechanical obstruction in the urinary passages, such as enlarged prostate, or urethral stricture, without fever, and without much impairment of digestion, and without the appearances of tubular casts or fat granules in the urine, under the microscope, it is highly probable that the albuminous quality of the urine is due to congestion of the corticle texture of the kidney, without structural change.

It is to this class of cases that the present patient belongs. He has had a stricture of the urethra for a considerable time.

The indications for treatment are, first, to remove the stricture by surgical means, which will be done by the Professor of Clinical Surgery, Prof. E. Andrews.

*Second*, to remove the congested condition of the kidneys, by increasing the tonicity of the capillary vessels and the quantity of urinary secretion. To accomplish this, he was directed the following formula:—

℞. Fl. Ext. Scutellaria, ---------------------- ℥ij.
Tinct. Mattico,------------------------------ ℥j.
Tinct. Digitalis, ---------------------------- ℥j.
Acetas Potassa,----------------------------- ʒiv.

Mix. Take one teaspoonful in water before each mealtime, and at bedtime.

Consumption. — The attention of the class was next called to a man of middle age, of strong muscular frame, with a cough. The Prof. examined his chest, anteriorly and posteriorly, and explained carefully the nature of the sounds to be heard by auscultation and percussion; after which each member of the class used the stethoscope.

Inspection showed full expansion of the left lung but constriction of the right. Percussion shows greater dulness of the right than the left lung. Auscultation reveals, in the right lung, a strong, bubbling rale, or submucous ronchus, on inspiration, more distinct near the end of the act, and a sonorous expiration. In the left, a strong bronchial, or exaggerated respiratory murmur, showing an increased force of air necessary to carry on the functions of life, owing to the crippled condition of the right. The patient complained of soreness in both lungs and pain in coughing. The cough came on suddenly, about five months ago, and had continued uninterruptedly since. The sputa was now copious, thick, and yellow, enveloped in mucus, and never contained blood. In chronic, capillary bronchitis, the wheezing in inspiration and expiration is prolonged, and there is not dulness on percussion; but, in this case, the breath is short and right lung dense. In emphysema, the air-cells are dilated, causing great clearness on percussion, and labored expiration. If the bronchial tubes were blocked up, by exudation, or from pressure, as by an aneurism, enlarged gland, a portion of the lung would collapse, excluding all air sounds, and causing increased dulness on percussion. Neither has there been any history of pneumonia giving rise to infiltration and solidification, nor any of the phenomena of pleuritic effusion, nor any history which might lead one to suppose the formation of a cancerous mass in the lung. But there has been all the phenomena of tubercle. The patient complained of catching cold and continuing to cough. His health became impaired and digestion disturbed, out of proportion to the local lesions. Emaciation has steadily progressed. Breathing growing shorter. The cough has become more harassing, by night and day. There is fever in the afternoon, with sweats at night. Pulse 100 in the afternoon, but only 80 in the morning. The cause of this fatal scourge is attributed to some peculiar property of the blood, which gives rise to congestion and the formation of a caco-plastic or partially organized substance called tubercle. At first, the deposits are isolated. After a time, they gradually accumulate and lead to more or less consolidation of the part. As the disease advances, these soften, and the surrounding living tissue takes on suppurative action, which gives rise to the sounds just now heard.

The indications for treatment, in the second or suppurative stage, which is the one presented by the patient before us, are, to allay the local pulmonary irritation, to correct coincident functional derangements, and to establish a more healthy and active assimilation and nutrition.

In the active, softening stage of pulmonary tuberculosis, many cases are accompanied by pain and soreness in some part of the chest, severe cough, and fever, especially in the afternoon and evening.

In such cases, the first indication named is one of much importance, because, in allaying the local pulmonary irritation, we not only lessen pain and soreness, but we greatly retard the destruction of tissue and limit the amount of suppuration. In the great majority of cases, the following prescription will fulfil this indication better than any other that we have tried:—

| | | |
|---|---|---|
| ℞. | Ammonia Hydrochlor., | ʒiij. |
| | Ant. et Pot. Tartras, | 2 grs. |
| | Morph. Sulph., | 3 grs. |
| | Glyzarrhiza Syrup, | ℥iv. |

Mix. Take one teaspoonful each morning, noon, teatime, and bedtime.

The most common coincident derangements are those connected with the digestive organs, such as deficient secretion of gastric juice, and consequent flatulency, constipation, and occasionally diarrhœa. The lecturer stated, that such disorders, connected with phthisis, in any stage of its progress, should receive careful attention. To make digestion more perfect, when the gastric juice is deficient, he recommended a teaspoonful of the pepsin glycerole (a preparation containing the active principle of the rennet in glycerine), immediately after each meal. To obviate constipation, one of the following pills may be taken each evening:—

℞. Ext. Hyosciamus, }

Sulph. Ferri, } āā,------------------ 30 grs.

Pulv. Aloes, }

Blue Mass,------------------------------ 15 grs.

Mix. Divide 30 Pills.

To promote nutrition, and thereby sustain the strength, and diminish the tendency to further tuburcular deposits, passive exercise in a mild, dry air, as much as possible, plain, simple nourishment, and the use of such articles as bismuth and iron, Liebig's ext. malt, etc., are the best means within our reach.

